# Effect of metronidazole on concentrations of vaginal bacteria associated with risk of HIV acquisition

**DOI:** 10.1128/mbio.01110-24

**Published:** 2024-11-21

**Authors:** D. J. Valint, Tina L. Fiedler, Congzhou Liu, Sujatha Srinivasan, David N. Fredricks

**Affiliations:** 1 Vaccine and Infectious Disease Division, Fred Hutchinson Cancer Center, Seattle, Washington, USA; 2 Department of Medicine, University of Washington, Seattle, Washington, USA; Washington University School of Medicine, St. Louis, Missouri, USA; Washington University School of Medicine, St. Louis, Missouri, USA

**Keywords:** vaginal microbiota, bacterial vaginosis, HIV risk, metronidazole, quantitative PCR

## Abstract

Several bacterial vaginosis (BV)-associated bacteria have been associated with elevated risk of human immunodeficiency virus (HIV) acquisition; however, susceptibility of these bacteria to antibiotics is poorly understood. Vaginal samples were collected from 22 persons daily for 2 weeks following BV diagnosis. Metronidazole treatment was prescribed for 5–7 days. Changes in bacterial concentrations were measured with taxon-specific 16S rRNA gene quantitative PCR (qPCR) assays. A culture-based antimicrobial assay confirmed presence of antibiotics in vaginal swab samples. Bacterial DNA concentrations decreased during antibiotic administration for all 13 bacterial taxa tested. Comparison of bacterial DNA concentrations in samples before administration of antibiotics to samples taken on the last day of antimicrobial assay-confirmed antibiotic presence showed a 2.25–4.78 log10-fold decrease across all taxa. Concentrations were frequently reduced to the qPCR assay’s limit of detection, suggesting eradication of bacteria. Mean clearance time varied across taxa (1.2–7.9 days), with several bacteria (e.g., *Sneathia* spp., Vaginal TM7, and *Eggerthella*-like sp.) taking >7 days to suppress. Metronidazole reduces quantities of bacterial taxa associated with increased HIV acquisition risk.

**IMPORTANCE:**

Human immunodeficiency virus (HIV) transmission through sex remains a major public health challenge despite efforts at risk reduction and use of anti-retroviral pre-exposure prophylaxis. Many bacterial vaginosis (BV)-associated vaginal bacteria have been associated with increased HIV infection risk among women. If these bacteria help mediate HIV infection risk, then eradication of these bacteria is one potential strategy to reduce this risk. However, the best approach to eradicate HIV-high risk bacteria from the vagina is not known. We analyzed vaginal swabs collected daily from women with BV to determine the impact of metronidazole treatment on 13 vaginal bacterial taxa linked to elevated risk of HIV infection through use of taxon-directed quantitative PCR assays. We conclude that eradication of high-risk vaginal bacteria using metronidazole is one promising avenue for reducing HIV acquisition risk, and we provide evidence that a 5–7-day treatment course may not be sufficient to suppress all bacteria.

## INTRODUCTION

Bacterial vaginosis (BV) is a gynecologic condition characterized by a shift in the vaginal microbiota in which the healthy *Lactobacillus*-dominated community is replaced by a complex and heterogeneous consortium of anaerobic bacteria (1–4). This shift in the microbial community is associated with several adverse sequelae, including an increased risk of sexually transmitted infections such as human immunodeficiency virus (HIV) (5, 6). A broad array of vaginal bacterial species has been associated with elevated risk of subsequent HIV acquisition, including *Prevotella* spp., *Sneathia* spp., *Parvimonas* spp., *Gemelliphila asaccharolytica* (previously *Gemella asaccharolytica* [7]), *Mycoplasma hominis*, *Porphyromonas* species Type 1, *Eggerthella*-like vaginal bacterial species Type 1, *Megasphaera* spp., *Amygdalobacter indicium* (previously BV-associated bacterium 2 [8]), *Peptoniphilus lacrimalis*, and Vaginal TM7 (9–11). Bacterial associations with HIV risk may be due to increased genital inflammation caused by bacteria (9, 11, 12), disruption of the cervicovaginal mucus layer by bacterial sialidases and mucinases (13, 14), or other factors. Regardless, identifying an effective intervention that can target these bacteria would allow for the assessment of the direct impact of bacteria on HIV acquisition and would provide a critical step toward devising additional preventive measures to mitigate HIV infection risk if bacteria play a causal role.

Current Centers for Disease Control and Prevention guidelines recommend metronidazole as first-line treatment for BV, though clindamycin, secnidazole, and tinidazole may also be used (15, 16). Metronidazole is often the preferred option due to its efficacy and low cost (17–19) and administered as a topically applied vaginal gel or orally ingested pills. Metronidazole is an antibiotic prodrug activated via reduction under anaerobic conditions and targets many anaerobic bacteria present in BV. Metronidazole treatment can have a dramatic effect on the vaginal bacterial community, resulting in a widespread reduction in bacterial concentrations across the diverse community of BV-associated bacteria (20). For some vaginal bacterial taxa such as *Gardnerella* spp. and *Fannyhessea vaginae*, metronidazole was effective in reducing the concentrations but not eradicating these bacteria (21, 22).

For many of the HIV risk-associated vaginal bacterial species, susceptibility to antibiotics is poorly documented. Many of these organisms are fastidious making direct measurements of *in vitro* antibiotic susceptibility difficult. Moreover, there is a paucity of *in vivo*, longitudinal studies of the vaginal microbiota that utilize quantitative PCR techniques to assess absolute changes in concentrations of vaginal bacterial species with antibiotic therapy. Based on an NCBI PubMed search with the search terms “(vaginal) AND ((microbiome) OR (microbiota)) AND (metronidazole) AND ((quantitative PCR) OR (qPCR)),” as of October 2023, only seven studies reported longitudinal, *in vivo* quantitative PCR (qPCR) data describing the shifts in vaginal bacterial concentrations over the course of metronidazole treatment (3, 22–27). Of these, only four included data on the concentrations of at least one bacterial taxon associated with HIV-risk (3, 22, 24, 26), and only two collected daily vaginal samples subjected to qPCR (3, 22). While the findings of Srinivasan et al. (3) and Mayer et al. (22) help elucidate the dynamic behavior of the vaginal bacterial community during and following antibiotic treatment for BV, both studies included a relatively small number of participants with BV and neither study specifically sought to clarify how HIV risk-associated bacterial taxa respond to metronidazole treatment.

To fill this knowledge gap, we leveraged data from a longitudinal study of persons with BV treated with metronidazole to determine how concentrations of vaginal bacteria change *in vivo* in response to antibiotic therapy, circumventing the need for laboratory cultivation to assess susceptibility. We targeted 13 vaginal bacterial species previously linked to elevated HIV infection risk and measured concentrations of bacterial DNA by qPCR in a longitudinal sample set of vaginal swabs collected daily for a 2-week period following BV diagnosis, capturing the entire duration of treatment, and approximately 1 week following cessation. Although *Gardnerella* spp. have not been strongly linked to HIV risk, the genus has a longstanding association with BV and thus was also included in our analysis. Our immediate goal was to determine whether metronidazole leads to eradication of high-risk vaginal bacteria, with an ultimate goal of identifying an intervention that could be studied to deplete these bacteria and assess impact on HIV infection risk.

## RESULTS

Thirty-two treatment courses were documented in 22 persons with BV. The mean age of all participants was 31.7 years (SD 6.2). Fifty-five percent of participants self-identified as White, and 41% as Black. All participants self-identified as non-Hispanic. Oral metronidazole was prescribed for 17 total treatment courses, while vaginal metronidazole gel was prescribed for 15. The median Nugent score prior to antibiotic treatment was 8, and scores ranged from 4 to 10 (Table 1), with clinical diagnosis of BV made by Amsel criteria.

**TABLE 1:** Description of study population

Characteristic	Value (*n* = 22)
Age, mean (SD), years	32 (6.2)
Race	
White	12 (54.6%)
Black	9 (40.9%)
Other	1 (4.6%)
Ethnicity	
Hispanic	0 (0%)
Non-Hispanic	22 (100%)
Treatment courses	(*n* = 32)
Oral metronidazole (7 d)	17
Vaginal metronidazole (5 d)	15
Nugent score prior to treatment, median (min.–max.)	8 (4–10)

An internal amplification control (IAC) PCR assay using jellyfish DNA to test for the presence of PCR inhibitors did not detect PCR inhibition in any of the samples, allowing for confidence in the accuracy of subsequent qPCR results. No-template PCR controls were reproducibly negative highlighting the lack of cross-well contamination during qPCR.

**Fig 1:**
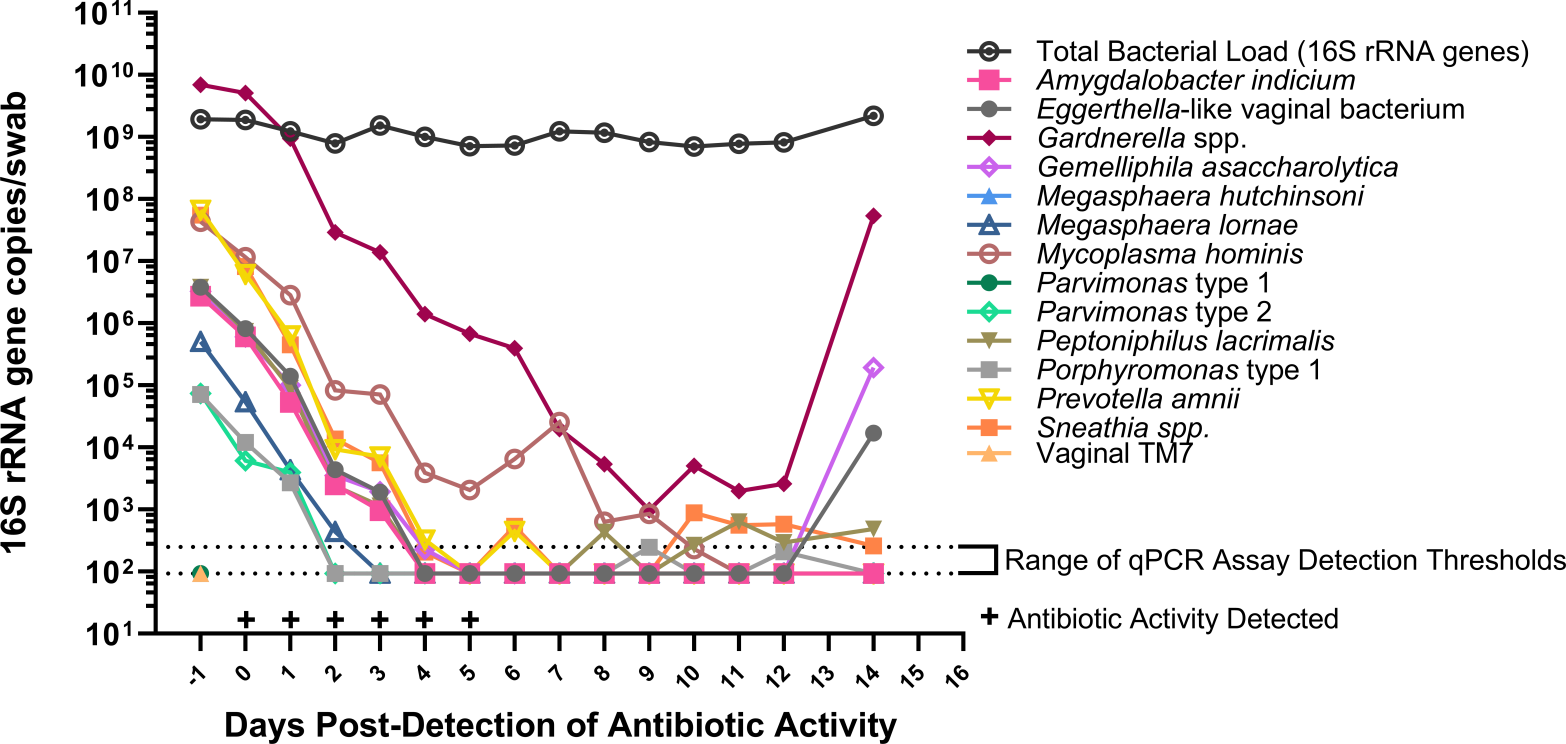
Concentrations of vaginal bacteria over time during metronidazole treatment for a representative treatment course in one person with BV.

**Fig 2:**
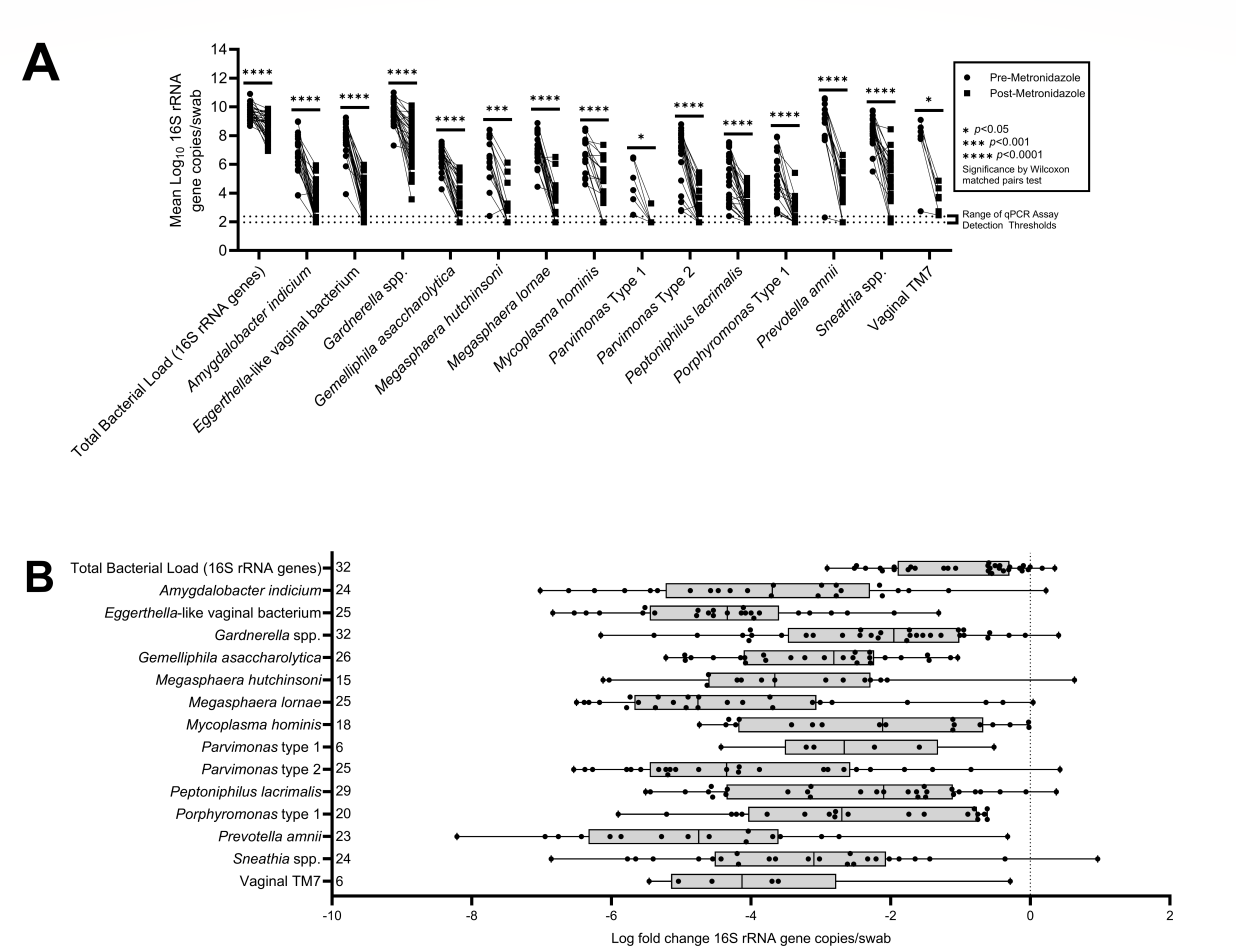
Two visualizations of the same data set, illustrating changes in concentrations of 16S rRNA gene copies per swab, comparing “pre-metronidazole” samples (baseline measurements collected 1 day prior to confirmed antibiotic presence) to “post-metronidazole” samples (collected on the final day of antibiotic activity detection). (A) Graph showing decreases in concentrations of bacterial DNA for each taxon assayed, from “pre-metronidazole” to “post-metronidazole” samples. Statistical significance assessed by Wilcoxon matched pairs test (Table S1). (B) Box plot displaying log-fold change in bacterial concentrations, from “pre-metronidazole” to “post-metronidazole” samples. Total number of treatments in which a bacterial taxon appeared is indicated to the right of each bacterial taxon.

Mean vaginal bacterial DNA concentrations decreased over the duration of antibiotic administration for all bacterial taxa tested, as reflected both by broad-range and taxon-specific qPCR assays and by documenting use of antibiotics in vaginal fluid by antimicrobial assay (Fig. 1 and 2).

Comparison of bacterial DNA concentrations from samples taken before administration of antibiotics to samples taken on the last day of assay-confirmed antibiotic presence showed multiple log10-fold decreases in bacterial DNA across all taxa. Total bacterial load was observed to decrease by 1.01 logs, on average; however, larger decreases were observed for more specific taxon groups. The smallest decrease in bacterial DNA concentration for a particular taxon was observed for *Mycoplasma hominis*, which only decreased by 2.25 logs on average. In the other extreme, we observed an average decrease in *Prevotella amnii* bacterial DNA concentration of 4.78 logs (Fig. 2).

In most cases, bacterial DNA concentrations were reduced to the assay’s limit of detection, suggesting bacterial eradication, though we cannot exclude continued presence of these bacteria at concentrations below our detection threshold. Suppression (defined as the observed reduction of concentration of 16S rRNA gene copies to the lower limit of detection by qPCR assay at any point during the 15-day window) was noted in 13 of the 14 bacterial taxa monitored. Suppression of *Gardnerella* spp. was not observed in any treatment course. For those taxa in which suppression was observed, the frequency of suppression varied from 58.33% (*Sneathia* spp., suppression reached in only 14 of 24 treatment courses) to 100% (Vaginal TM7, suppression reached in 6 of 6 treatment courses) (Table 2).

**TABLE 2:** Suppression of high-risk bacterial taxa with metronidazole treatment

Bacterial taxa	Total treatments present, no. (%)	Treatments reaching suppression within 2 weeks, no. (%)
*Amygdalobacter indicium*	24 (75.0)	18 (75.0)
*Eggerthella*-like vaginal bacterium	25 (78.1)	19 (76.0)
*Gardnerella* spp.	32 (100.0)	0 (0.0)
*Gemelliphila asaccharolytica*	26 (81.3)	19 (73.1)
*Megasphaera hutchinsoni*	15 (46.9)	12 (80.0)
*Megasphaera lornae*	25 (78.1)	21 (84.0)
*Mycoplasma hominis*	18 (56.3)	11 (61.1)
*Parvimonas* Type 1	6 (18.8)	5 (83.3)
*Parvimonas* Type 2	25 (78.1)	21 (84.0)
*Peptoniphilus lacrimalis*	29 (90.6)	25 (86.2)
*Porphyromonas* Type 1	20 (62.5)	18 (90.0)
*Prevotella amnii*	16 (50.0)	12 (75.0)
*Sneathia* spp.	24 (75.0)	14 (58.3)
Vaginal TM7	6 (18.8)	6 (100.0)

**Fig 3:**
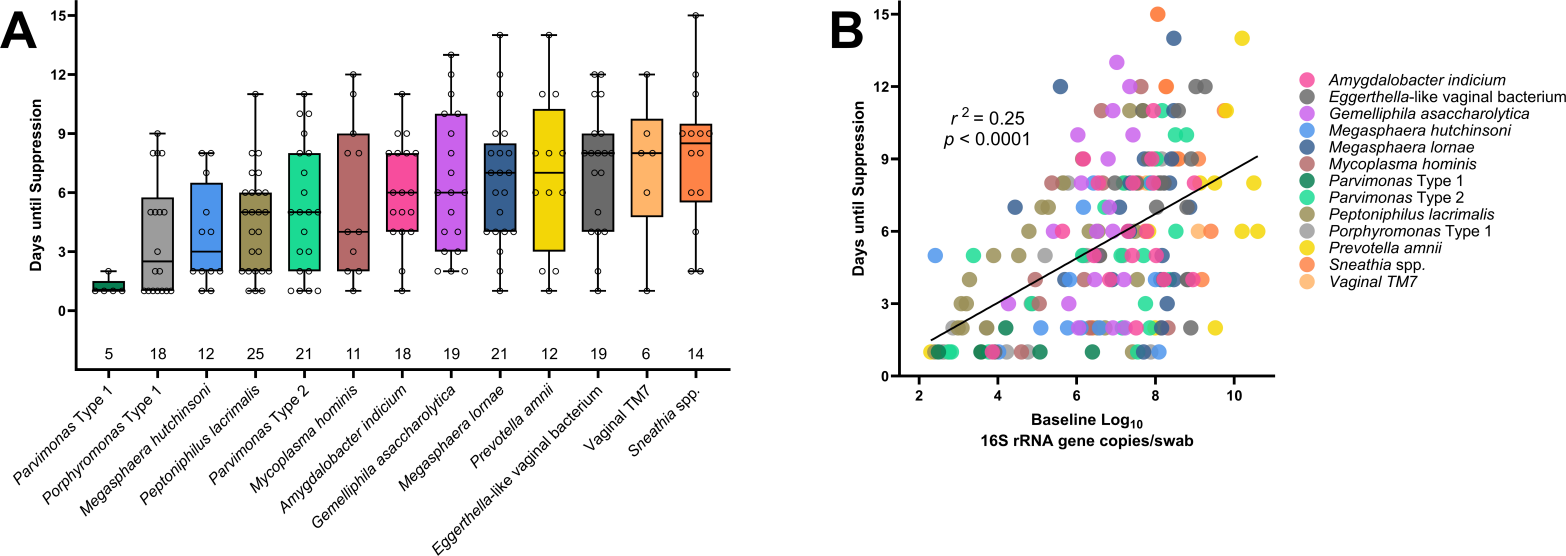
Box plot (**A**) showing time to suppression for each bacterial taxon (total number of treatments in which a bacterial taxon appeared is indicated along base of x-axis); scatter plot (**B**) showing relationship between baseline bacterial concentration and time to suppression.

Mean time to suppression varied between taxa. *Parvimonas* Type 1 was one of the most rapidly suppressed taxa, requiring on average only 1.2 days to reach suppression, while *Sneathia* spp. required the longest time to suppress (average of 7.86 days). Three taxa (*Eggerthella*-like sp., *Sneathia* spp., and Vaginal TM7) required on average greater than 7 days to reach suppression (Fig. 3A). Higher baseline bacterial load was correlated with longer time until suppression [r(204)=0.497, p<0.001], though the strength of the correlation was low (*r*^2^ = 0.25) (Fig. 3B).

**Fig 4:**
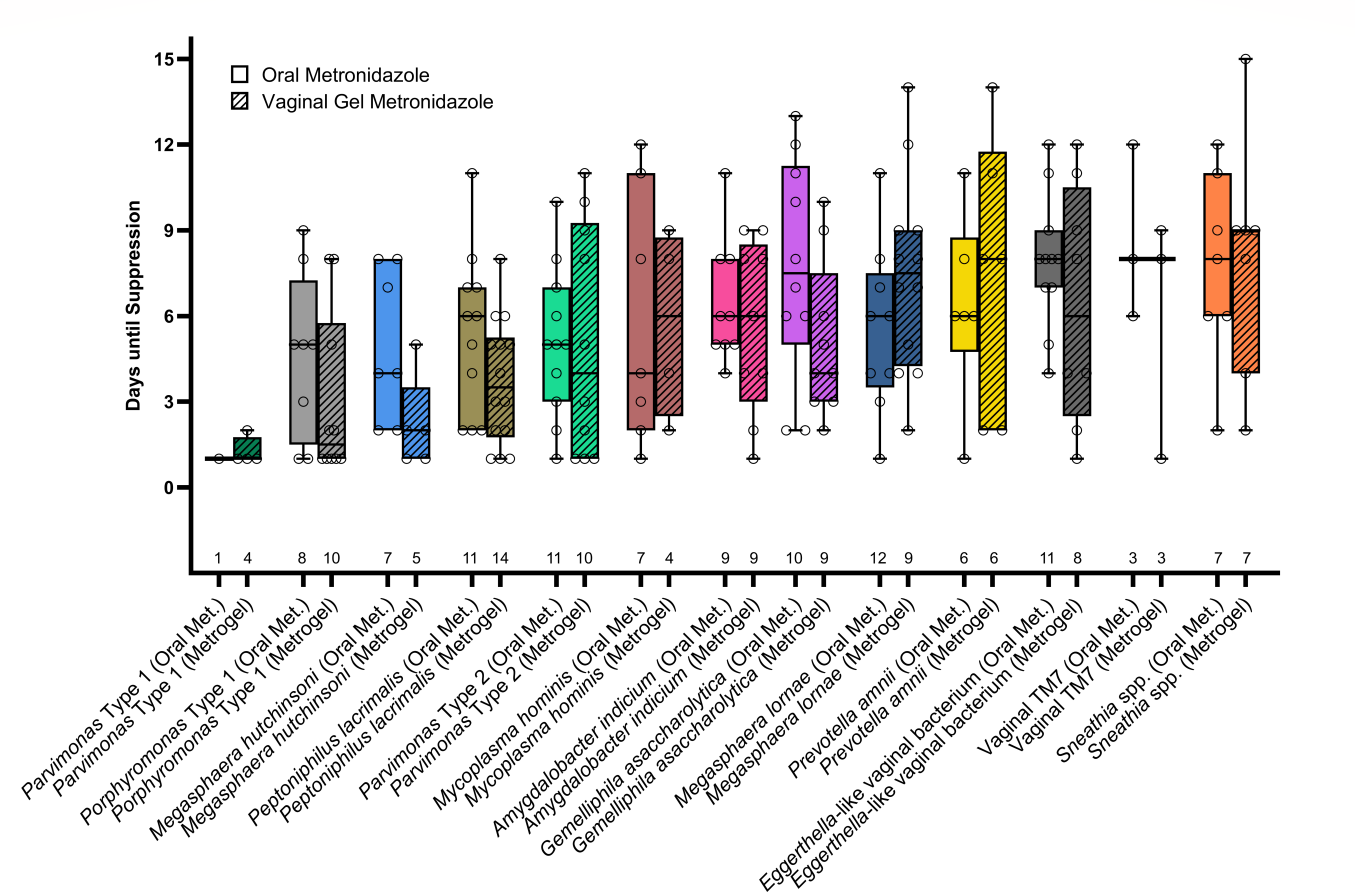
Box plot showing time to suppression between different bacterial taxa, split by method of antibiotic administration (total number of treatments in which a bacterial taxon appeared is indicated along base of x-axis). Statistical significance assessed by Mann-Whitney U-test (Table S2).

No significant differences were observed in time to suppression between orally administered (7-day) vs. topically applied (5-day) metronidazole for any of the assayed taxa (Fig. 4).

## DISCUSSION

In this study, we demonstrate that the administration of metronidazole for treatment of BV reduced concentrations of 14 vaginal bacterial taxa previously associated with increased risk of HIV acquisition and other negative vaginal health outcomes as measured by taxon-specific qPCR assays. While all 14 taxa were found to decrease in concentration over the period of treatment, the magnitude of response of each taxon varied. *Parvimonas* Type 1, *Porphyromonas* Type 1, and *Megasphaera hutchinsoni* were most rapidly depleted, reaching the threshold for suppression in an average of <4 days. In contrast, *Eggerthella*-like sp., *Sneathia* spp., and Vaginal TM7 were observed to persist for a longer duration, detected above the threshold for suppression for >7 days on average. Moderate decreases in concentration were observed for *Gardnerella* spp., but concentrations were never observed to reach levels consistent with our definition of suppression. Total bacterial load was observed to decrease over the duration of antibiotic treatment, but only minimally. This may be due to increases in concentrations of taxa not investigated in this study (i.e., *Lactobacillus* spp.) concurrent with the decreases in concentrations observed for the 14 taxa assayed. Our findings suggest that while metronidazole is effective in reducing the concentrations of bacterial taxa linked to increased risk of HIV acquisition, a 5–7-day treatment course of metronidazole may not be sufficient to fully suppress all taxa, including *Eggerthella*-like sp., *Sneathia* spp., and Vaginal TM7. Our observation of the failure of metronidazole treatment to effectively suppress *Gardnerella* spp. is consistent with the findings in reference (22), in which they demonstrated that metronidazole therapy is not effective for *Gardnerella* eradication. Due to the high degree of similarity in the 16S rRNA gene sequences of *Gardnerella* species, the qPCR primers utilized in this study to measure *Gardnerella* concentrations were only capable of genus-level resolution. However, different *Gardnerella* strains have been shown to exhibit differences in susceptibility to metronidazole (28, 29). This 5–7-day antibiotic duration has been found effective in treating acute BV. However, the likelihood of recurrence of BV after treatment is high, with observed recurrence rates of up to 80% (21, 30, 31). The exact cause of recurrent BV is not fully understood and is likely multifactorial, but one potential factor may be the failure of a standard treatment course of metronidazole to completely eradicate all pathogenic bacterial taxa. The results of this study support that hypothesis and indicate that for several vaginal bacterial taxa associated with BV and HIV-acquisition, the standard of care treatment is insufficient for suppression of bacteria to levels undetectable by qPCR. As such, a longer duration of treatment or more bactericidal antibiotics may be required to eradicate some vaginal bacteria. However, continuation of antibiotic treatment beyond the standard of care has not been observed to be effective at reducing BV recurrence (18) though initial investigations of high-dose vaginal metronidazole therapy have shown encouraging results (32, 33).

Though this study focused on taxa previously shown to be associated with elevated HIV risk, our results may be relevant when considering other adverse BV-associated health outcomes, such as pre-term birth, pelvic inflammatory disease, and increased acquisition risk for other sexually transmitted infections such as with *Neisseria gonorrhoeae*, *Chlamydia trachomatis*, and *Trichomonas vaginalis* (34–38). Effective eradication of BV-associated bacteria may have ramifications for decreasing risk of these and other BV-associated health outcomes.

Metronidazole was observed to be equally effective in suppressing bacterial concentrations when administered orally or as a vaginally applied gel. In a 2009 study, Mitchell et al. compared oral and vaginal metronidazole for the treatment of BV in pregnancy and reported comparable changes in concentrations of most bacterial taxa as evaluated by qPCR (39). Notably, *Sneathia* spp. showed greater response to oral treatment; however, we did not note this difference in the present study. This discrepancy may be explained by the low number of individuals with presence of *Sneathia* spp. in our study (vaginal *n* = 7).

This study reflects a dense longitudinal analysis of the vaginal microbiota during antibiotic therapy using quantitative PCR to illustrate how several vaginal bacterial taxa associated with increased HIV infection risk respond to metronidazole treatment. The high degrees of sensitivity, specificity, and dynamic range for the qPCR assays allow for a high degree of confidence when declaring a bacterial taxon to have been suppressed in a given treatment course. Additionally, the longitudinal nature of the sample set allowed for the capture of data points before, during, and following the antibiotic treatment window.

Another strength of this study is the ability to accurately assess antibiotic adherence compared with self-report. An objective test to measure antibiotic presence in a sample allowed us to confirm a participant’s adherence to antibiotic treatment and therefore increase confidence in our conclusions. Additionally, for this study, we measured antibiotic response *in vivo*, reflecting biologically relevant concentrations of antibiotic.

A limitation of our study is the modestly sized study population; however, we had sufficient power to demonstrate successful eradication of most bacteria. The longitudinal sample set used in this study was limited to 15 days following the onset of treatment for BV; thus, we did not capture the long-term dynamics of the bacterial communities following metronidazole therapy. Furthermore, our definition of suppression does not address the issue of recurrence—it only addresses the capacity for and rate at which metronidazole therapy reduces the concentrations of bacterial taxa to the limits of detection by each respective assay. In terms of the study population, this cohort was comprised of a roughly even split of White and Black persons with a history of BV, located in the Seattle, WA area. As such, the results of this study may not be generalizable to other populations, such as African populations in which bacteria-HIV risk associations have been more thoroughly documented. qPCR measures bacterial DNA concentrations, and the presence of bacterial DNA may not reflect viable bacteria, though the absence of bacterial DNA does imply that concentrations of bacterial cells are low or absent.

We conclude that metronidazole therapy was effective at decreasing vaginal concentrations of 13 bacterial taxa associated with increased risk of HIV acquisition, although the time required for suppression often exceeded 7 days for several taxa. Eradication of high-risk vaginal bacteria using metronidazole is one promising avenue to explore for reducing risk for HIV acquisition.

## MATERIALS AND METHODS

### Study population, treatment, and sampling

Between October 2012 and June 2016, persons with BV were enrolled in a study of the vaginal microbiota conducted by the University of Washington and the Fred Hutchinson Cancer Center in Seattle, WA. Protocols were approved by the Fred Hutchinson Institutional Review Board (IRB Protocol #7683), and all study participants provided written informed consent. For each participant with BV, antibiotic treatment with metronidazole was prescribed either as 5 g of vaginally applied gel (with 37.5 mg of metronidazole) daily for 5 days or orally ingested pills of 500 mg twice daily for 7 days. BV was diagnosed using Amsel clinical criteria (40). Daily vaginal swabs encompassing a 15-day window beginning immediately prior to antimicrobial assay-confirmed initiation of antibiotic treatment were collected spanning 32 individual antibiotic treatment courses from 22 different persons with BV. Nugent scores based on vaginal fluid Gram stain (41) prior to antibiotic treatment were calculated using the baseline vaginal samples to also assess for BV.

### Confirmation of antibiotic use

To evaluate adherence to antibiotics, an antimicrobial test was developed to confirm the presence of antibiotics in vaginal swab samples. This assay used vaginal fluid inoculated onto plates with cultured bacteria susceptible to metronidazole. Vaginal swabs were washed by vortex mixing in cold, filtered saline (4°C, Millipore Amicon Ultra-15 100 kDa MWCO Centrifugal Filter) for 2 minutes. Resulting fluid was then centrifuged at 14,000 rpm for 10 minutes at 4°C to separate pellet. Supernatant was removed and added into holes punctured in agar plates prepared with a lawn of bacteria. Fifty microliters of supernatant was inoculated onto plates prepared with *Prevotella amnii*, and 100 µL of supernatant was inoculated onto plates prepared with *Megasphaera hutchinsoni*. Presence of antibiotics in the supernatant was indicated by the development of a ring of clearance in the bacterial lawn around the punctured holes in the agar after incubation for 22–24 hours at 37°C under anaerobic conditions.

Samples from a treatment course were only included for further analysis if antibiotic presence was observed in at least 80% of samples (a minimum of 4 out of 5 days) if prescribed vaginal metronidazole gel or 6 out of 7 days if prescribed oral metronidazole starting from the first day of observed presence of antibiotics.

### Measurement of concentrations of individual bacterial taxa

DNA was extracted from vaginal swabs using a QIAamp BiOstic Bacteremia DNA Isolation Kit and subjected to taxon-specific 16S rRNA gene qPCR assays to monitor changes in concentrations of 14 bacterial taxa, each of which has been shown to be associated with elevated HIV infection risk (10, 11). Previously published assays were employed targeting the 16S rRNA gene for *Amygdalobacter indicium* (BVAB2), *Megasphaera lornae*, *Sneathia* spp. (20), *Eggerthella-*like sp., *Prevotella amnii* (42), *Gemella asaccharolytica*, *Mycoplasma hominis*, *Parvimonas* sp. Type 1, *Parvimonas* sp. Type 2, *Porphyromonas* sp. Type 1 (10), Vaginal TM7 (43), *Megasphaera hutchinsoni* (44), and *Peptoniphilus lacrimalis* (11). Total bacterial load was similarly assessed using a broad-range qPCR assay targeting the bacterial 16S rRNA gene (4). To test for the presence of PCR inhibitors, an IAC PCR assay using jellyfish DNA was conducted on all samples (45). No-template negative controls were included for each qPCR assay.

### Definition of suppression

For each bacterial taxon, if the concentration of bacterial DNA reached the lower limit of detection for the qPCR assay during the 15-day sampling window, that taxon was defined as “suppressed” for that treatment course. Lower limits of detection ranged from 93.75 to 187.5 16S rRNA gene copies/swab, varying between taxa on the basis of assay sensitivity. The number of “days until suppression” was counted starting from the baseline day (1–3 days prior to confirmed antibiotic presence, as determined by sample availability) until the first day for which qPCR data qualified a taxa/treatment course as “suppressed.”

### Data analysis

To assess the significance of observed decreases in concentrations of bacterial DNA from baseline, Wilcoxon-matched pairs tests were conducted for each taxon. Correlation between baseline concentrations of bacterial DNA and time required to suppression was evaluated by Pearson’s correlation coefficient. Observed differences in time to suppression between orally administered vs. topically applied metronidazole gel were assessed for statistical significance by Mann-Whitney U-test. All statistical analyses and data visualization were performed using GraphPad Prism 10.2.3.

## Data Availability

Antibiotic test results for each sample and qPCR data set are provided in the supplemental material.

## SUPPLEMENTAL MATERIAL

Data Set S1 mbio.01110-24-s0001.xlsx Antibiotic test and qPCR assay data.

Figure S1 mbio.01110-24-s0002.tif Bacterial concentrations over time for each treatment course.

Figure S2 mbio.01110-24-s0003.tif Bacterial concentrations over time for each taxon.

Supplemental Legends mbio.01110-24-s0004.docx Legends for Fig. S1 and S2.

Table S1 mbio.01110-24-s0005.tif *P* values for Fig. 2A.

Table S2 mbio.01110-24-s0006.tif *P* values for Fig. 4.

ASM does not own the copyrights to Supplemental Material that may be linked to, or accessed through, an article. The authors have granted ASM a non-exclusive, world-wide license to publish the Supplemental Material files. Please contact the corresponding author directly for reuse.

## References

[1] Fredricks DN, Fiedler TL, Marrazzo JM. 2005. Molecular identification of bacteria associated with bacterial vaginosis. N Engl J Med 353:1899–1911. doi:10.1056/NEJMoa043802 16267321

[2] Ravel J, Brotman RM, Gajer P, Ma B, Nandy M, Fadrosh DW, Sakamoto J, Koenig SS, Fu L, Zhou X, Hickey RJ, Schwebke JR, Forney LJ. 2013. Daily temporal dynamics of vaginal microbiota before, during and after episodes of bacterial vaginosis. Microbiome 1:29. doi:10.1186/2049-2618-1-29 24451163 PMC3968321

[3] Srinivasan S, Liu C, Mitchell CM, Fiedler TL, Thomas KK, Agnew KJ, Marrazzo JM, Fredricks DN. 2010. Temporal variability of human vaginal bacteria and relationship with bacterial vaginosis. PLoS One 5:e10197. doi:10.1371/journal.pone.0010197 20419168 PMC2855365

[4] Srinivasan S, Hoffman NG, Morgan MT, Matsen FA, Fiedler TL, Hall RW, Ross FJ, McCoy CO, Bumgarner R, Marrazzo JM, Fredricks DN. 2012. Bacterial communities in women with bacterial vaginosis: high resolution phylogenetic analyses reveal relationships of microbiota to clinical criteria. PLoS One 7:e37818. doi:10.1371/journal.pone.0037818 22719852 PMC3377712

[5] Cohen CR, Lingappa JR, Baeten JM, Ngayo MO, Spiegel CA, Hong T, Donnell D, Celum C, Kapiga S, Delany S, Bukusi EA. 2012. Bacterial vaginosis associated with increased risk of female-to-male HIV-1 transmission: a prospective cohort analysis among African couples. PLoS Med 9:e1001251. doi:10.1371/journal.pmed.1001251 22745608 PMC3383741

[6] Martin HL, Richardson BA, Nyange PM, Lavreys L, Hillier SL, Chohan B, Mandaliya K, Ndinya-Achola JO, Bwayo J, Kreiss J. 1999. Vaginal lactobacilli, microbial flora, and risk of human immunodeficiency virus type 1 and sexually transmitted disease acquisition. J Infect Dis 180:1863–1868. doi:10.1086/315127 10558942

[7] Bello S, Mudassir SH, Rudra B, Gupta RS. 2023. Phylogenomic and molecular markers based studies on Staphylococcaceae and Gemella species. Proposals for an emended family Staphylococcaceae and three new families (Abyssicoccaceae fam. nov., Salinicoccaceae fam. nov. and Gemellaceae fam. nov.) harboring four new genera, Lacicoccus gen. nov., Macrococcoides gen. nov., Gemelliphila gen. nov., and Phocicoccus gen. nov. Antonie Van Leeuwenhoek 116:937–973. doi:10.1007/s10482-023-01857-6 37523090

[8] Srinivasan S, Austin MN, Fiedler TL, Strenk SM, Agnew KJ, Gowda GAN, Raftery D, Beamer MA, Achilles SL, Wiesenfeld HC, Fredricks DN, Hillier SL. 2023. Amygdalobacter indicium gen. nov., sp. nov., and Amygdalobacter nucleatus sp. nov., gen. nov.: novel bacteria from the family Oscillospiraceae isolated from the female genital tract. Int J Syst Evol Microbiol 73:006017. doi:10.1099/ijsem.0.006017 37787404 PMC11318147

[9] Gosmann C, Anahtar MN, Handley SA, Farcasanu M, Abu-Ali G, Bowman BA, Padavattan N, Desai C, Droit L, Moodley A, Dong M, Chen Y, Ismail N, Ndung’u T, Ghebremichael MS, Wesemann DR, Mitchell C, Dong KL, Huttenhower C, Walker BD, Virgin HW, Kwon DS. 2017. Lactobacillus-deficient cervicovaginal bacterial communities are associated with increased HIV acquisition in young South African women. Immunity 46:29–37. doi:10.1016/j.immuni.2016.12.013 28087240 PMC5270628

[10] McClelland RS, Lingappa JR, Srinivasan S, Kinuthia J, John-Stewart GC, Jaoko W, Richardson BA, Yuhas K, Fiedler TL, Mandaliya KN, Munch MM, Mugo NR, Cohen CR, Baeten JM, Celum C, Overbaugh J, Fredricks DN. 2018. Evaluation of the association between the concentrations of key vaginal bacteria and the increased risk of HIV acquisition in African women from five cohorts: a nested case-control study. Lancet Infect Dis 18:554–564. doi:10.1016/S1473-3099(18)30058-6 29396006 PMC6445552

[11] Srinivasan S, Richardson BA, Wallis JM, Fiedler TL, Strenk SM, Hoffman NG, Proll S, Chirenje ZM, Livant EW, Fredricks DN, Hillier SL, Marrazzo JM. 2024. Vaginal bacteria and proinflammatory host immune mediators as biomarkers of human immunodeficiency virus acquisition risk among African women. J Infect Dis:jiae406. doi:10.1093/infdis/jiae406 39248500 PMC11646615

[12] Anahtar MN, Byrne EH, Doherty KE, Bowman BA, Yamamoto HS, Soumillon M, Padavattan N, Ismail N, Moodley A, Sabatini ME, Ghebremichael MS, Nusbaum C, Huttenhower C, Virgin HW, Ndung’u T, Dong KL, Walker BD, Fichorova RN, Kwon DS. 2015. Cervicovaginal bacteria are a major modulator of host inflammatory responses in the female genital tract. Immunity 42:965–976. doi:10.1016/j.immuni.2015.04.019 25992865 PMC4461369

[13] Cauci S, Culhane JF. 2011. High sialidase levels increase preterm birth risk among women who are bacterial vaginosis-positive in early gestation. Am J Obstet Gynecol 204:142. doi:10.1016/j.ajog.2010.08.061 21055720

[14] McGregor JA, French JI, Jones W, Milligan K, McKinney PJ, Patterson E, Parker R. 1994. Bacterial vaginosis is associated with prematurity and vaginal fluid mucinase and sialidase: results of a controlled trial of topical clindamycin cream. Am J Obstet Gynecol 170:1048–1059; doi:10.1016/s0002-9378(94)70098-2 8166188

[15] Weir CB, Le JK. 2023. Metronidazole. StatPearls [Internet]. Treasure Island (FL): StatPearls Publishing. Available from: https://www.ncbi.nlm.nih.gov/books/NBK539728/?report=classic

[16] Workowski KA, Bachmann LH, Chan PA, Johnston CM, Muzny CA, Park I, Reno H, Zenilman JM, Bolan GA. 2021. Sexually transmitted infections treatment guidelines, 2021. MMWR Recomm Rep 70:1–187. doi:10.15585/mmwr.rr7004a1 PMC8344968 34292926

[17] Schmitt C, Sobel JD, Meriwether C. 1992. Bacterial vaginosis: treatment with clindamycin cream versus oral metronidazole. Obstet Gynecol 79:1020–1023. 1579299

[18] Schwebke JR, Desmond RA. 2007. A randomized trial of the duration of therapy with metronidazole plus or minus azithromycin for treatment of symptomatic bacterial vaginosis. Clin Infect Dis 44:213–219. doi:10.1086/509577 17173219

[19] Sobel JD. 1997. Vaginitis. N Engl J Med 337:1896–1903. doi:10.1056/NEJM199712253372607 9407158

[20] Fredricks DN, Fiedler TL, Thomas KK, Mitchell CM, Marrazzo JM. 2009. Changes in vaginal bacterial concentrations with intravaginal metronidazole therapy for bacterial vaginosis as assessed by quantitative PCR. J Clin Microbiol 47:721–726. doi:10.1128/JCM.01384-08 19144794 PMC2650913

[21] Bradshaw CS, Tabrizi SN, Fairley CK, Morton AN, Rudland E, Garland SM. 2006. The association of Atopobium vaginae and Gardnerella vaginalis with bacterial vaginosis and recurrence after oral metronidazole therapy. J Infect Dis 194:828–836. doi:10.1086/506621 16941351

[22] Mayer BT, Srinivasan S, Fiedler TL, Marrazzo JM, Fredricks DN, Schiffer JT. 2015. Rapid and profound shifts in the vaginal microbiota following antibiotic treatment for bacterial vaginosis. J Infect Dis 212:793–802. doi:10.1093/infdis/jiv079 25676470 PMC4539900

[23] Ahrens P, Andersen LO, Lilje B, Johannesen TB, Dahl EG, Baig S, Jensen JS, Falk L. 2020. Changes in the vaginal microbiota following antibiotic treatment for Mycoplasma genitalium, Chlamydia trachomatis and bacterial vaginosis. PLoS One 15:e0236036. doi:10.1371/journal.pone.0236036 32722712 PMC7386580

[24] Armstrong E, Hemmerling A, Miller S, Burke KE, Newmann SJ, Morris SR, Reno H, Huibner S, Kulikova M, Liu R, Crawford ED, Castañeda GR, Nagelkerke N, Coburn B, Cohen CR, Kaul R. 2022. Metronidazole treatment rapidly reduces genital inflammation through effects on bacterial vaginosis–associated bacteria rather than lactobacilli. J Clin Invest 132:e152930. doi:10.1172/JCI152930 35113809 PMC8920324

[25] Armstrong E, Hemmerling A, Miller S, Burke KE, Newmann SJ, Morris SR, Reno H, Huibner S, Kulikova M, Nagelkerke N, Coburn B, Cohen CR, Kaul R. 2022. Sustained effect of LACTIN-V (Lactobacillus crispatus CTV-05) on genital immunology following standard bacterial vaginosis treatment: results from a randomised, placebo-controlled trial. Lancet Microbe 3:e435–e442. doi:10.1016/S2666-5247(22)00043-X 35659905 PMC9188188

[26] Balkus JE, Srinivasan S, Anzala O, Kimani J, Andac C, Schwebke J, Fredricks DN, McClelland RS. 2017. Impact of periodic presumptive treatment for bacterial vaginosis on the vaginal microbiome among women participating in the preventing vaginal infections trial. J Infect Dis 215:723–731. doi:10.1093/infdis/jiw622 28007924 PMC5853550

[27] van de Wijgert JHHM, Verwijs MC, Agaba SK, Bronowski C, Mwambarangwe L, Uwineza M, Lievens E, Nivoliez A, Ravel J, Darby AC. 2020. Intermittent lactobacilli-containing vaginal probiotic or metronidazole use to prevent bacterial vaginosis recurrence: a pilot study incorporating microscopy and sequencing. Sci Rep 10:3884. doi:10.1038/s41598-020-60671-6 32127550 PMC7054572

[28] Schuyler JA, Mordechai E, Adelson ME, Sobel JD, Gygax SE, Hilbert DW. 2016. Identification of intrinsically metronidazole-resistant clades of Gardnerella vaginalis. Diagn Microbiol Infect Dis 84:1–3. doi:10.1016/j.diagmicrobio.2015.10.006 26514076

[29] Landlinger C, Oberbauer V, Podpera Tisakova L, Schwebs T, Berdaguer R, Van Simaey L, Vaneechoutte M, Corsini L. 2022. Preclinical data on the Gardnerella-specific endolysin PM-477 indicate Its potential to improve the treatment of bacterial vaginosis through enhanced biofilm removal and avoidance of resistance. Antimicrob Agents Chemother 66:e0231921. doi:10.1128/aac.02319-21 35416708 PMC9112913

[30] Bradshaw CS, Sobel JD. 2016. Current treatment of bacterial vaginosis-limitations and need for innovation. J Infect Dis 214 Suppl 1:S14–S20. doi:10.1093/infdis/jiw159 27449869 PMC4957510

[31] Vodstrcil LA, Muzny CA, Plummer EL, Sobel JD, Bradshaw CS. 2021. Bacterial vaginosis: drivers of recurrence and challenges and opportunities in partner treatment. BMC Med 19:194. doi:10.1186/s12916-021-02077-3 34470644 PMC8411528

[32] Aguin T, Akins RA, Sobel JD. 2014. High-dose vaginal maintenance metronidazole for recurrent bacterial vaginosis: a pilot study. Sex Transm Dis 41:290–291. doi:10.1097/OLQ.0000000000000123 24722380

[33] Sobel JD, Kaur N, Woznicki NA, Boikov D, Aguin T, Gill G, Akins RA. 2019. Conventional oral and secondary high dose vaginal metronidazole therapy for recurrent bacterial vaginosis: clinical outcomes, impacts of sex and menses. Infect Drug Resist 12:2297–2307. doi:10.2147/IDR.S213853 31413606 PMC6661983

[34] Baisley K, Changalucha J, Weiss HA, Mugeye K, Everett D, Hambleton I, Hay P, Ross D, Tanton C, Chirwa T, Hayes R, Watson-Jones D. 2009. Bacterial vaginosis in female facility workers in north-western Tanzania: prevalence and risk factors. Sex Transm Infect 85:370–375. doi:10.1136/sti.2008.035543 19473997 PMC2709714

[35] Bautista CT, Wurapa EK, Sateren WB, Morris SM, Hollingsworth BP, Sanchez JL. 2017. Association of bacterial vaginosis with chlamydia and gonorrhea among women in the U.S. Army. Am J Prev Med 52:632–639. doi:10.1016/j.amepre.2016.09.016 27816380

[36] Gallo MF, Macaluso M, Warner L, Fleenor ME, Hook EW III, Brill I, Weaver MA. 2012. Bacterial vaginosis, gonorrhea, and chlamydial infection among women attending a sexually transmitted disease clinic: a longitudinal analysis of possible causal links. Ann Epidemiol 22:213–220. doi:10.1016/j.annepidem.2011.11.005 22192490

[37] Leitich H, Bodner-Adler B, Brunbauer M, Kaider A, Egarter C, Husslein P. 2003. Bacterial vaginosis as a risk factor for preterm delivery: a meta-analysis. Am J Obstet Gynecol 189:139–147. doi:10.1067/mob.2003.339 12861153

[38] Wiesenfeld HC, Hillier SL, Krohn MA, Amortegui AJ, Heine RP, Landers DV, Sweet RL. 2002. Lower genital tract infection and endometritis: insight into subclinical pelvic inflammatory disease. Obstet Gynecol 100:456–463. doi:10.1016/s0029-7844(02)02118-x 12220764

[39] Mitchell CM, Hitti JE, Agnew KJ, Fredricks DN. 2009. Comparison of oral and vaginal metronidazole for treatment of bacterial vaginosis in pregnancy: impact on fastidious bacteria. BMC Infect Dis 9:89. doi:10.1186/1471-2334-9-89 19515236 PMC2703644

[40] Amsel R, Totten PA, Spiegel CA, Chen KCS, Eschenbach D, Holmes KK. 1983. Nonspecific vaginitis. Diagnostic criteria and microbial and epidemiologic associations. Am J Med 74:14–22. doi:10.1016/0002-9343(83)91112-9 6600371

[41] Nugent RP, Krohn MA, Hillier SL. 1991. Reliability of diagnosing bacterial vaginosis is improved by a standardized method of gram stain interpretation. J Clin Microbiol 29:297–301. doi:10.1128/jcm.29.2.297-301.1991 1706728 PMC269757

[42] Srinivasan S, Morgan MT, Fiedler TL, Djukovic D, Hoffman NG, Raftery D, Marrazzo JM, Fredricks DN. 2015. Metabolic signatures of bacterial vaginosis. MBio 6:e00204-15. doi:10.1128/mBio.00204-15 25873373 PMC4453549

[43] Haggerty CL, Ness RB, Totten PA, Farooq F, Tang G, Ko D, Hou X, Fiedler TL, Srinivasan S, Astete SG, Fredricks DN. 2020. Presence and concentrations of select bacterial vaginosis-associated bacteria are associated with increased risk of pelvic inflammatory disease. Sex Transm Dis 47:344–346. doi:10.1097/OLQ.0000000000001164 32149953 PMC7167351

[44] Sabo MC, Lokken EM, Srinivasan S, Kinuthia J, Richardson BA, Fiedler TL, Munch M, Proll S, Salano C, John-Stewart G, Jaoko W, Fredricks DN, McClelland RS. 2023. Changes in vaginal bacteria and inflammatory mediators from periconception through early-postpartum in a cohort of HIV-negative Kenyan women. J Infect Dis 228:487–499. doi:10.1093/infdis/jiad168 37207618 PMC10428199

[45] Khot PD, Ko DL, Hackman RC, Fredricks DN. 2008. Development and optimization of quantitative PCR for the diagnosis of invasive aspergillosis with bronchoalveolar lavage fluid. BMC Infect Dis 8:73. doi:10.1186/1471-2334-8-73 18510764 PMC2440748

