# Supplementary material for: Effect of metronidazole on concentrations of vaginal bacteria associated with risk of HIV acquisition
Source: mBio. 2024 Nov 21;15(12):e01110-24. doi: 10.1128/mbio.01110-24 (PMC11633388; doi:10.1128/mbio.01110-24)
Supplement: Supplemental Legends — Legends for Fig. S1 and S2. [file mbio.01110-24-s0004.docx]

**Supplemental Figure Legends**

**Figure S1.** Concentrations of vaginal bacteria over time during metronidazole treatment for each treatment course.

**Figure S2.** Concentrations of vaginal bacteria over time during metronidazole treatment for each bacterial taxon across all treatment courses.
