# Supplementary figures and images for: Effect of metronidazole on concentrations of vaginal bacteria associated with risk of HIV acquisition
Source: mBio. 2024 Nov 21;15(12):e01110-24. doi: 10.1128/mbio.01110-24 (PMC11633388; doi:10.1128/mbio.01110-24)

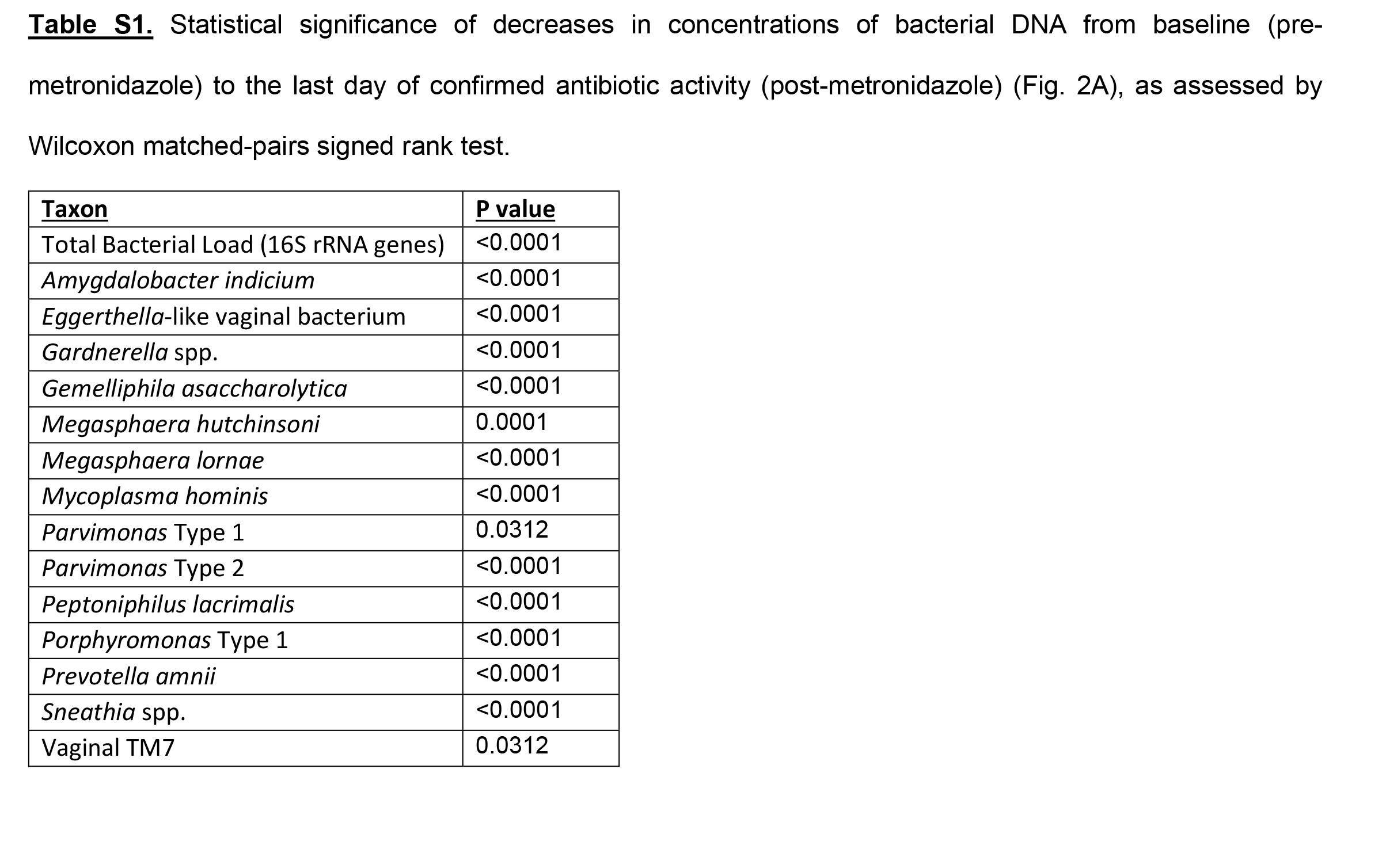

Supplement: Table S1 — P values for Fig. 2A. [file mbio.01110-24-s0005.tif]

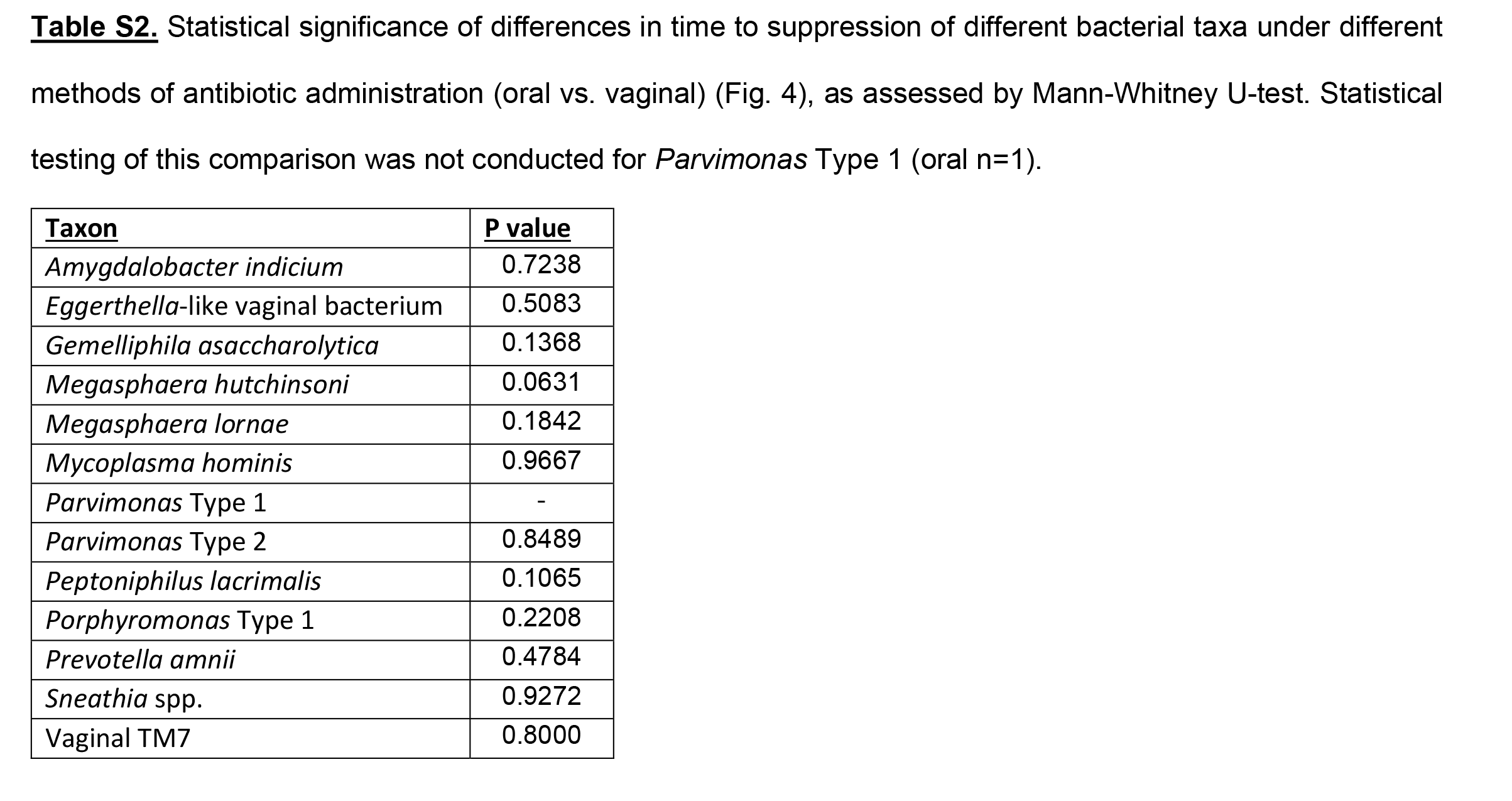

Supplement: Table S2 — P values for Fig. 4. [file mbio.01110-24-s0006.tif]
